# Duplicate left gastric artery identified during robot-assisted distal gastrectomy: a case report

**DOI:** 10.1186/s40792-023-01698-5

**Published:** 2023-08-23

**Authors:** Hikota Hayashi, Noriyuki Hirahara, Takeshi Matsubara, Satoshi Takao, Hiroki Okamura, Kosuke Nakamura, Takashi Kishi, Takahito Taniura, Hitomi Zotani, Kazunari Ishitobi, Yoshitsugu Tajima

**Affiliations:** Department of Digestive and General Surgery, Shimane University Faculty of Medicine, 89-1, Enya-Cho, Izumo, Shimane 693-8501 Japan

**Keywords:** Duplicate left gastric artery, Gastrectomy, Gastric cancer

## Abstract

**Background:**

Duplicated left gastric artery (LGA) is a rare anomaly. With an incidence of only 0.4%, its clinical significance remains largely unrecognized.

**Case presentation:**

A 65-year-old man underwent robot-assisted distal gastrectomy for early gastric cancer. After division of the left gastric vein in the left gastropancreatic fold, a slim LGA (LGA-1) was identified and dissected. Careful dissection of the left gastropancreatic fold toward the root of the celiac artery revealed another LGA (LGA-2), which was dissected without difficulty. Postoperative reevaluation of the three-dimensional-computed tomography (CT) angiography reconstructed using the preoperative CT scan identified a 2.7 mm LGA-1, branching from the splenic artery, and a 3.0 mm LGA-2, branching from the celiac artery. To the best of our knowledge, this is only the third reported case of a duplicate LGA in a patient who underwent laparoscopic gastrectomy. Our case is the first to report the use of robot surgery.

**Conclusions:**

Although duplicate LGA is rare and receives little clinical attention, surgeons should keep this vascular anomaly in mind during preoperative evaluation since there is an increased risk for intraoperative bleeding during gastrectomy.

## Background

When performing gastrectomy with lymph node dissection for gastric cancer, thorough understanding of the anatomy of the abdominal arteries and veins, including its potential vascular anomalies, is crucial to ensure surgical safety. The anatomical classification of the upper abdominal vessels, such as the Adachi classification [1], enables surgeons to understand vascular anomalies preoperatively [1, 2].

A duplicated left gastric artery (LGA) is an anomaly of the celiac artery. In a review of 500 patients who underwent celiac angiography, Naidich et al. [3] reported the incidence of a duplicate LGA to be 0.4% (two cases). Only two cases of duplicate LGAs identified during gastrectomy have been documented [4, 5]. Although duplicate LGA may be negligible in frequency, and little attention has been paid to this anomaly, unexpected intraoperative bleeding during gastrectomy has been reported [4]. We report a case of duplicate LGA identified during robot-assisted distal gastrectomy (RDG) for early gastric cancer, where the vascular anomaly was not recognized during preoperative evaluation. Careful intraoperative observation, with an attentive surgical maneuver, allowed us to safely manage the two LGAs.

## Case presentation

A 65-year-old man, with early-stage gastric cancer, was referred to our hospital. He was 170 cm tall and weighed 67 kg (body mass index of 23.1 kg/m^2^). He was asymptomatic and had an unremarkable medical history. Endoscopic biopsy of the depressed gastric tumor (type 0-IIc + III) [6] revealed a well-to-moderately differentiated adenocarcinoma. However, submucosal involvement was observed on endoscopic ultrasonography. No nodal or distant metastases were noted on contrast-enhanced abdominal computed tomography (CT). Serum levels of CA19-9 and carcinoembryonic antigen were within normal limits. The patient was diagnosed with early-stage gastric cancer, clinical stage I (T1bN0M0) [7], and was scheduled to undergo RDG with regional lymph node dissection.

**Fig. 1:**
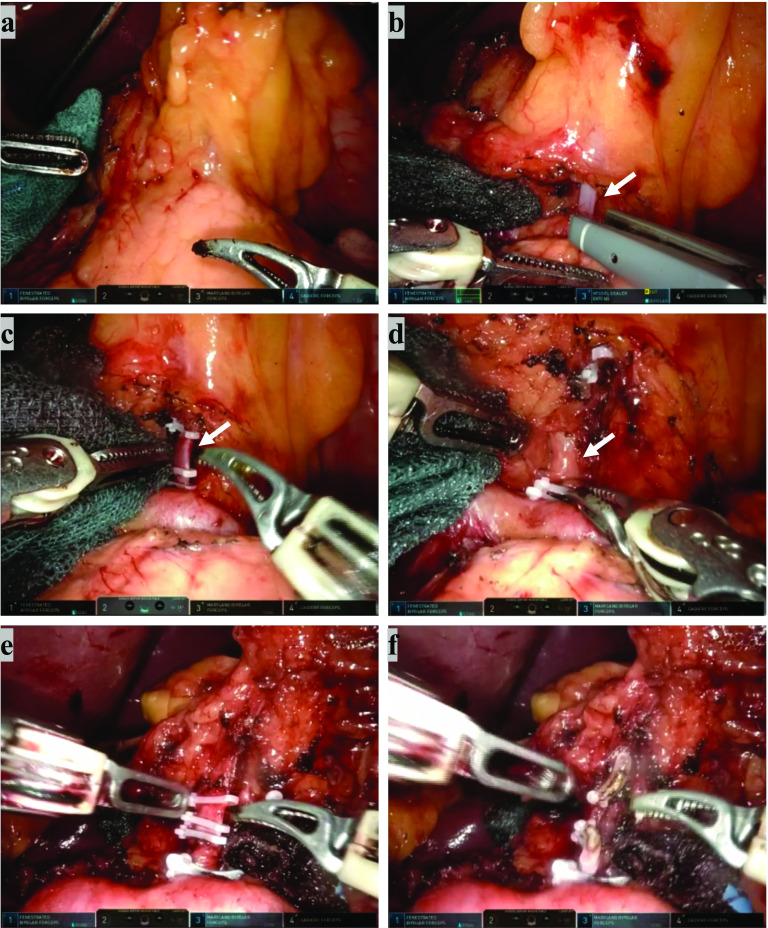
Intraoperative view during robot-assisted distal gastrectomy. **a** The left gastropancreatic fold. **b** Dissection of the left gastric vein (arrow) using a vessel sealer. **c** Double clips and dissection of the first identified left gastric artery (arrow) (LGA-1). **d** Exposure of the left gastric artery (arrow) identified after dissection of LGA-1 (LGA-2). **e** Double clips and dissection of LGA-2. **f** After completion of the left gastric vein, LGA-1, and management of LGA-2

The da Vinci Xi Surgical System (Intuitive Surgical, Inc. Tokyo, Japan) was used. After exposing the left gastropancreatic fold (Fig. 1a), the left gastric vein was identified, clipped, and divided using a vessel-sealing device (Fig. 1b). Behind the left gastric vein, the LGA-1 was identified, doubly clipped, and divided following artery denervation. Because the LGA-1 appeared to be slightly more slender than usual (Fig. 1c), careful dissection was carried out toward the root of the celiac artery, where the duplicate LGA (LGA-2) was identified (Fig. 1d). The two LGAs were similar in size, and ran towards the lesser curvature of the stomach. The LGA-2 was exposed carefully along the outermost layer for lymphadenectomy, denervated, doubly clipped, and divided (Fig. 1e, f). LGA-1 and LGA-2 both branched individually and had different nerve plexuses The RDG was completed without any complications. The patient’s postoperative course was uneventful, and he was discharged from the hospital on postoperative day 8.

**Fig. 2:**
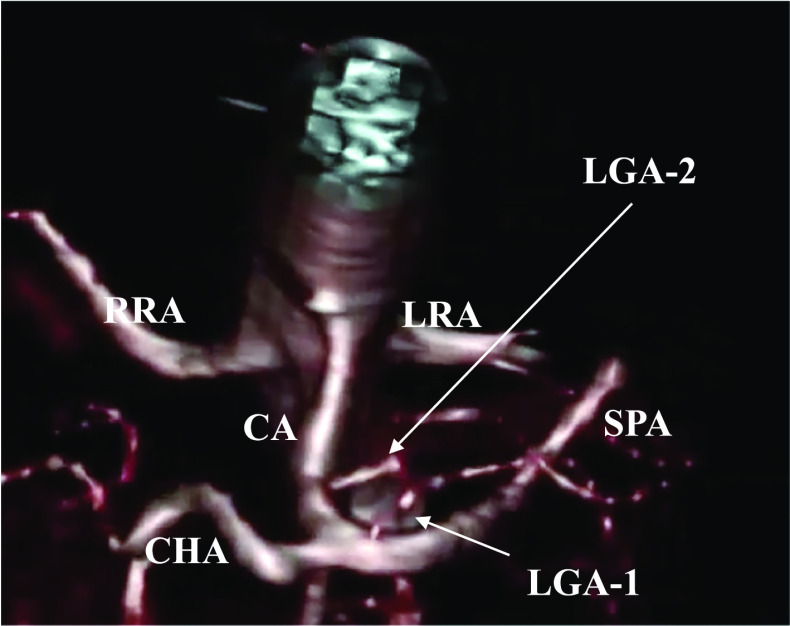
Caudal view of the three-dimensional-computed tomography angiogram demonstrating the LGA-1 branching from the root of the splenic artery and the LGA-2 arising from the celiac artery. *CA* celiac artery, *CHA* common hepatic artery, *SPA* splenic artery, *RRA* right renal artery, *LRA* left renal artery, *LGA* left gastric artery

Postoperatively, we reevaluated the preoperative contrast-enhanced abdominal CT scan and identified two LGAs. Both arteries fed the lesser curvature of the stomach, LGA-1 towards the oral side and LGA-2 towards the anal side. A three-dimensional (3D)-CT angiogram with multiplanar reconstruction using the Synapse Vincent 4.0 (Fuji Film, Japan) revealed that the LGA-1, 2.7 mm in diameter, branched from the root of the splenic artery and the LGA-2, 3.0 mm diameter, branched from the main trunk of the celiac artery (Fig. 2).

## Discussion

Although the incidence of duplicate LGA is reported to be 0.4% [3], surgeons may encounter this vascular anomaly during gastrectomy owing to the large number of gastrectomies performed for various gastroenterological disorders. Several well-known texts on upper abdomen arterial variations, such as the Adachi’s [1] and Michels’ [2] classifications, are available. However, there is no description of a duplicate LGA, possibly due to its rarity and lack of clinical attention. However, Taki et al. [4] previously reported the first case of a duplicate LGA, identified during laparoscopic distal gastrectomy for early-stage gastric cancer, in which the anomalous artery was discovered after unexpected pulsating bleeding was observed from a small vessel in the nerve plexus around the LGA (Table 1). In our case, the presence of the duplicate LGA was not seen during the preoperative evaluation. However, postoperative reconstruction of the 3D-CT angiography from the preoperative CT data clearly showed LGA-1 branching from the splenic artery and LGA-2 branching from the celiac artery (Fig. 2). Fortunately, we were able to identify LGA-2 immediately after handling LGA-1, as the LGA-1 was noted to be somewhat smaller than usual. Had we failed to note this, the LGA-2 may have otherwise been divided during lymphadenectomy along the celiac artery. Although recent energy devices can seal vessels up to 7 mm in diameter [8], duplicate LGAs should be recognized as a potential risk for unexpected bleeding during gastrectomy. An aberrant left hepatic artery (LHA), originating from the LGA and running to the left lobe of the liver through the gastrohepatic ligament, is commonly encountered during gastrectomy [9, 10]. Urabe et al. [5] previously reported a case of duplicate LGA in a patient with early gastric cancer, in whom preoperative 3D-CT angiography identified both an aberrant LHA arising from the LGA and a duplicate LGA originating from the celiac trunk, thus contributing to successful laparoscopic gastrectomy (Table 1). The authors emphasized the need for a preoperative simulation of the vascular anatomy, using 3D-CT angiography, because vascular anatomies, including duplicate LGA, carry a possible risk of causing life-threatening morbidities during abdominal surgery.

**Table 1:** Characteristics of the duplicate left gastric artery identified during gastrectomy

Author	Location and maximum diameter (mm) of duplicate LGA	Preoperative recognition of duplicate LGA	Intraoperative events
Taki et al. [4]	LGA-1: celiac artery (1.2 mm) LGA-2: celiac artery (2.5 mm)	No	LGA-1 injury
Urabe et al. [5]	LGA-1: celiac artery (NA) LGA-2: aberrant LHA( NA)	Yes	None
Present case	LGA-1: splenic artery (2.7 mm) LGA-2: celiac artery (3.0 mm)	No	None

*LGA-1* left gastric artery firstly identified during surgery

*LGA-2* left gastric artery secondary identified during surgery

*NA* not available

When duplicate LGAs are in close proximity to each other and surrounded by the same nerve plexus, it may be difficult to distinguish the LGA from other small vessels, as is in the case by Taki et al. If we had experienced a case like this with robotic surgery, we would have been left to visual judgment and might have found ourselves in the same situation. Thus, close attention should be paid to unexpected bleeding. In contrast, in cases where the duplicate LGAs are distant from each other, surrounded by individual nerve plexus or a complicated branch, as in this report and that by Urabe et al., technical difficulty during lymph node dissection may be observed. However, as long as careful and meticulous manipulation is done, robotic surgery has no significant disadvantages. Instead, it allows a multidirectional approach to the anatomical structures of interest. Indeed, a meta-analysis showed that a greater number of lymph nodes are dissected during robotic surgery [11].

Despite the increased use of laparoscopic and robot-assisted surgeries for various gastric disorders, little attention has been paid to duplicate LGAs. Gastrointestinal surgeons should keep in mind all factors that potentially affect patient safety [12, 13], including duplicate LGAs. Currently, a systematic classification of duplicate LGAs and their exact definition has not been established. From a surgical perspective, we propose that a duplicate LGA is defined as an artery that is sufficiently large to be recognized intraoperatively and requires hemostasis, including prophylactic modalities such as clipping and coagulation. A precise classification of vascular anomalies, including duplicate LGA, based on further anatomical studies and imaging data, should be established.

## Conclusion

Duplicate LGA is a vascular anomaly that surgeons should be aware of preoperatively as a potential risk factor for unexpected bleeding during gastrectomy.

## Data Availability

All data are based on the medical records.

## Abbreviations

LGA: Left gastric artery
LGA-1: Left gastric artery first identified during surgery
LGA-2: Left gastric artery secondarily identified during surgery
RDG: Robot-assisted distal gastrectomy
LHA: Left hepatic artery
CT: Computed tomography
3D: Three-dimensional
